# Analysis of neutralizing antibodies to COVID-19 inactivated or subunit recombinant vaccines in hospitalized patients with liver dysfunction

**DOI:** 10.3389/fimmu.2023.1084646

**Published:** 2023-01-18

**Authors:** Hu Li, Shiyin Li, Pan Xu, Xiaohao Wang, Huan Deng, Yu Lei, Shan Zhong

**Affiliations:** Key Laboratory of Molecular Biology for Infectious Diseases (Ministry of Education), Institute for Viral Hepatitis, Department of Infectious Diseases, The Second Affiliated Hospital of Chongqing Medical University, Chongqing, China

**Keywords:** COVID-19, SARS-CoV-2, vaccine, neutralizing antibodies, liver dysfunction, artificial liver

## Abstract

**Background:**

The neutralizing antibodies (NAbs) response after COVID-19 vaccination after liver dysfunction is unclear. In this study, we evaluated the NAbs response after COVID-19 vaccination in hospitalized patients suffering from liver dysfunction.

**Methods:**

In this cross-sectional study with longitudinal follow-up, we enrolled eligible patients with liver dysfunction and healthy volunteers with full-course COVID-19 vaccination. Blood samples were collected for the NAbs testing at the time of admission and after treatment. Multiple regression analysis to assess independent risk factors affecting NAbs response.

**Results:**

A total of 137 patients and 134 healthy controls (HC) were enrolled. Both seropositivity (65.7% *vs* 80.6%, p<0.01) and titer (3.95 *vs* 4.94 log_2_ AU/ml, p<0.001) of NAbs in patients were significantly lower than that in HC. The decrease of antibody titer in patients was significantly faster than that in HC. After adjusting for potential confounding factors, males (odds ratio [OR]: 0.17; 95% confidence interval [CI]: 0.06, 0.46; p<0.001) and severe liver damage (OR: 0.30; 95% CI: 0.12, 0.71; p<0.01) were significantly associated with reduction of the probability of NAbs seropositivity in the multiple regression analysis. Males (β =-1.18; 95% CI: -1.73,-0.64) and chronic liver diseases (β =-1.45; 95% CI: -2.13, -0.76) were significantly associated with lower NAbs titers. In 26 patients with liver failure, both antibody seropositivity (53.8% *vs* 84.6%, p<0.05) and titer (3.55 *vs* 4.32 log_2_ AU/ml, p<0.001) did not decrease but increased after artificial liver plasmapheresis.

**Conclusions:**

NAbs response to COVID-19 inactivated or subunit recombinant vaccines was waning in patients with liver dysfunction. Moreover, patients with male sex, severe liver injury and chronic liver diseases have an increased risk of poor antibody responses.

## 1 Introduction

Coronavirus disease 2019 (COVID-19), caused by severe acute respiratory syndrome coronavirus 2 (SARS-CoV-2) infection, has been a life-threatening global health burden (1, 2). As of 23 October 2022, over 624 million confirmed cases and over 6.5 million deaths have been reported globally. Numerous studies have confirmed that SARS-CoV-2 vaccines can effectively reduce the morbidity and mortality of COVID-19 (3, 4). Vaccination against COVID-19 is currently considered the most effective, safe, and cost-effective way to prevent and control COVID-19 worldwide.

Previous studies have shown that patients with chronic liver disease are highly susceptible to COVID-19 and often have poor prognosis (5, 6). Therefore, some guidelines and expert consensus have been developed, and patients with chronic liver disease are encouraged to receive full-course SARS-CoV-2 vaccination (7, 8). Many previous studies have evaluated the safety and immunogenicity of SARS-CoV-2 vaccination in various chronic liver disease populations, including nonalcoholic fatty liver disease (9), post-liver transplant population (10), chronic hepatitis B (11), cirrhosis (12), and autoimmune liver disease (13, 14). These studies all showed the safety of SARS-CoV-2 vaccine in patients with liver disease, but the immune response to the vaccine was weaker than that in healthy people. The time after vaccination in these studies was too short for long-term observational data. In addition, some patients with chronic liver disease may experience acute liver damage due to various reasons. It is urgent for doctors to evaluate the antibody levels produced by prior COVID-19 immunizations after liver damage-caused by various reasons.

COVID-19 inactivated and receptor-binding domain (RBD) subunit recombinant vaccines are two common types of vaccines in China. However, whether the antibody responses following COVID-19 immunization is affected by liver injury is still unclear. In this study, we evaluated antibody responses in hospitalized patients with various causes of liver dysfunction after immunization with COVID-19 inactivated and RBD-subunit recombinant vaccines.

## 2 Patients and methods

### 2.1 Study design and participants

We did a cross-sectional study with longitudinal follow-up at the Second Affiliated Hospital of Chongqing Medical University from October 1, 2021, to January 30, 2022. The hospitalized patients with liver dysfunction were enrolled in the Center for Liver and Infectious diseases. At the same time, an equal number of healthy volunteers (healthy controls) who underwent annual physical examinations at the health management center were enrolled as controls. Inclusion criteria of participants were age 18 years or older, completed full-course of COVID-19 vaccination, no SARS-CoV-2 infection before receipt of the first vaccine dose (determined based on either a negative anti-SARS-CoV-2 immunoglobulin (Ig)-M/IgG test or the absence of a positive polymerase chain reaction (PCR) assay result for SARS-CoV-2, with no history of suspected clinical SARS-CoV-2 infection), and ability to understand and complete questionnaires in Chongqing, China. For healthy controls, no underlying disease history was also required. Participants with HIV co-infection, sepsis, cancers, severe extra-hepatic organ dysfunction, pregnancy during study entry, those who did not complete the full-course of vaccination, and those who provided incomplete vaccination information (including the date of their first vaccine dose and complete vaccination, and vaccine manufacturer [due to different vaccine types by manufacturers]) were excluded. All participants in this study provided about 5-10 microliters (mL) of peripheral blood samples for serological assays.

This study was approved by the Ethics Committee of the Second Affiliated Hospital of Chongqing Medical University. All participants signed an informed consent form.

### 2.2 Variable collection

We collected demographic characteristics (age and sex), history of SARS-CoV-2 infection (yes or no, results of anti-SARS-CoV-2 IgM/IgG and PCR assay for SARS-CoV-2 nucleic acid, and whether had clinical manifestations of SARS-CoV-2 infection), history of diseases, and vaccination-related information (the date of their first vaccine dose and complete vaccination, and vaccine types) in all participants utilizing a standardized questionnaire. Dates of vaccination and the types of vaccine were further confirmed by viewing the individual’s health code which contains complete vaccination-related information. Clinical parameters including laboratory test results and comorbidities were also collected from the electronic patient files in the hospital.

Full-course COVID-19 vaccination was defined as having had two vaccinations of inactivated vaccines or three vaccinations for the RBD-subunit recombinant vaccine. The interval time was defined as the days between the final dose and the first blood sample collection for serological assays. In the longitudinal analysis, we divided the time post-vaccination into three points, M1-2 (30-60 days), M3-4 (61-120 days), and >M4 (more than 120 days), respectively.

In patients receiving artificial liver therapy for liver failure, antibody testing was performed before and after treatment to assess the effect of artificial liver therapy on antibody levels. The treatment mode of artificial liver was plasma exchange combined with plasma adsorption.

### 2.3 SARS-CoV-2 antibody testing

Neutralizing antibodies (NAbs) testing was performed using the commercially available Novel Coronavirus Neutralizing Antibody Detection Kit (Chemiluminescence) (Shenzhen Yahuilong Biological Technology Co., LTD, Shenzhen, China) according to the manufacturer’s instructions in use. The cut-off value was determined to be 5.00 AU/mL.

### 2.4 Statistical analysis

Continuous variables are presented as means and standard deviations (SD) or as medians and interquartile ranges (IQR) for normally and nonnormally distributed data, respectively. Categorical variables are presented as percentages (%). NAbs titers were log2-transformed due to skewed distribution. To compare the distribution of NAbs seropositivity and titers, we used analysis of chi-square tests and Student t-test tests. We used paired t-tests to analyze NAbs titers before and after artificial liver treatment.

We used multiple regression analysis to characterize the association between variables and NAbs response. We adjusted for covariates using the crude model and full adjustment models. The crude model was unadjusted. The full adjustment model was adjusted for age (continuous variable), total bilirubin (continuous variable), cirrhosis (binary variable), ACLF (binary variable), vaccine types (binary variable), and interval time (continuous variable). Data are presented as point estimates and corresponding 95% confidence intervals (CI) of the effect size estimates.

All statistical analysis was performed using SPSS (version 24.0) and R 3.0 (http://www.R-project.org, the R Foundation). All statistical tests were two-sided, and p<0.05 was considered statistically significant.

## 3 Results

### 3.1 Participant characteristics

A total of 137 patients and 134 healthy controls (HC) were enrolled. **Table 1** presents the demographic characteristics, laboratory test results, comorbidities, and vaccination information of these participants. The mean age was 50.2 years (SD: 13.8 years) in patients and 42.6 years (SD:14.8 years) in HC. The proportions of males were 64.2% (88/137) and 38.1% (51/134) in patients and HC groups, respectively. The etiology of liver injury included 38.7% (53/137) of hepatitis B virus (HBV), 19.7% (27/137) of hepatitis C virus (HCV), 5.1% of hepatitis E virus (HEV), 9.5% (13/137) of autoimmune hepatitis, 6.6% (9/137) of alcohol, and 12.4% (17/137) of drugs. Of the 137 patients, 77.4% (106/137) had chronic liver diseases, 47.5% (65/137) had decompensated cirrhosis, and 48.2% (66/137) were diagnosed with acute-on-chronic liver failure (ACLF). The majority of patients and HC received the COVID-19 inactivated vaccine (82.5% *vs* 69.4%, respectively).

**Table 1:** Characteristics of the participants.

Variables	Patients (n=137)	Healthy controls (n=134)
Age (years), mean (SD)	50.2 (13.8)	42.6 (14.8)
Male, n (%)	88 (64.2)	51 (38.1)
ALT (U/L), median (IQR)	86.0 (40.0-266.0)	29.0 (20.0-35.0)
Total bilirubin (μmol/L), median (IQR)	71.0 (20.0-199.7)	17.1 (11.5-22.5)
Etiology, n (%)		
HBV	53 (38.7)	0 (0)
HCV	27 (19.7)	0 (0)
HEV	7 (5.1)	0 (0)
Autoimmune	13 (9.5)	0 (0)
Alcohol	9 (6.6)	0 (0)
Drug	17 (12.4)	0 (0)
Others	11 (8.0)	0 (0)
Chronic liver diseases, n (%)	106 (77.4)	0 (0)
Cirrhosis, n (%)	65 (47.5)	0 (0)
ACLF, n (%)	66 (48.2)	0 (0)
Vaccine type, n (%)		
Inactivated	113 (82.5)	93 (69.4)
RBD-subunit recombinant	24 (17.5)	41 (30.6)
Interval time (days), mean (SD) ^*^	118.8 (52.7)	91.7 (54.7)

Data are presented as mean (SD), median (IQR), or n (%). ACLF, acute on chronic liver failure; ALT, alanine aminotransferase; HBV, hepatitis B virus; HCV, hepatitis C virus; HEV, hepatitis E virus; IQR, interquartile range; RBD, receptor-binding domain; SD, standard deviation.

^*^Days between the final dose and first blood sample collection for serological assays.

### 3.2 Antibody responses after COVID-19 vaccination

**Figure 1:**
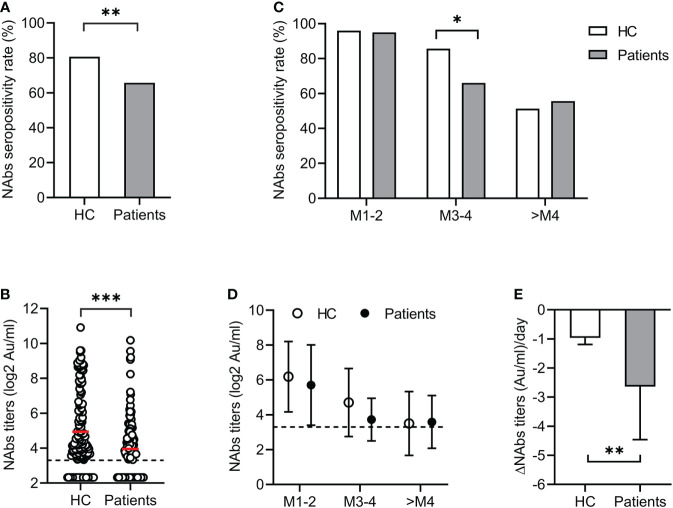
Antibody responses after COVID-19 vaccination. The seropositivity **(A)** and titers **(B)** of NAbs in patients with liver dysfunction and healthy controls (HC). Dynamic changes of NAbs seropositivity **(C)** and titers **(D)** over time during longitudinal follow-up. **(E)** Comparison of decline rate of NAbs titers in patients with liver dysfunction and HC groups. *<0.05; **<0.01; ***<0.001. AU, arbitrary units; COVID-19, coronavirus disease 2019; HC, healthy controls; NAbs, neutralizing antibodies.

In the cross-sectional study, the seropositivity for NAbs in patients was significantly lower than that in the HC group (65.7% *vs* 80.6%, p<0.01) (**Figure 1A**). The mean NAbs titer in patients was also lower than in the HC group (3.95 *vs* 4.94 log_2_AU/ml, p<0.001) (**Figure 1B**). During longitudinal follow-up, both seropositivity and titers of NAbs decreased over time in the patient and HC groups (**Figures 1C, D**). Between 3 and 4 months, the NAbs seropositivity in the patients decreased from 95.0% to 66.1%, while that in the HC group decreased from 96.0% to 85.7%. Therefore, the NAbs seropositivity in the patients was significantly lower than that in the HC group at M3-4 (p<0.05). Over time, only half of the participants were positive for NAbs, either in patients or in HC (55.7%, and 51.4% respectively) (**Figure 1C**). As for NAbs titers, the rate of decrease in patients was significantly faster than that in the HC group (-2.64 *vs* -0.97 AU/ml per day, p<0.01) (**Figure 1E**). Our results showed that the NAbs responses were impaired in patients with liver dysfunction. In addition, these patients have less persistence of antibody response than the HC group within 3-4 months after vaccination.

### 3.3 Risk factors for poor antibody response after COVID-19 vaccination in patients with liver injury

**Figure 2:**
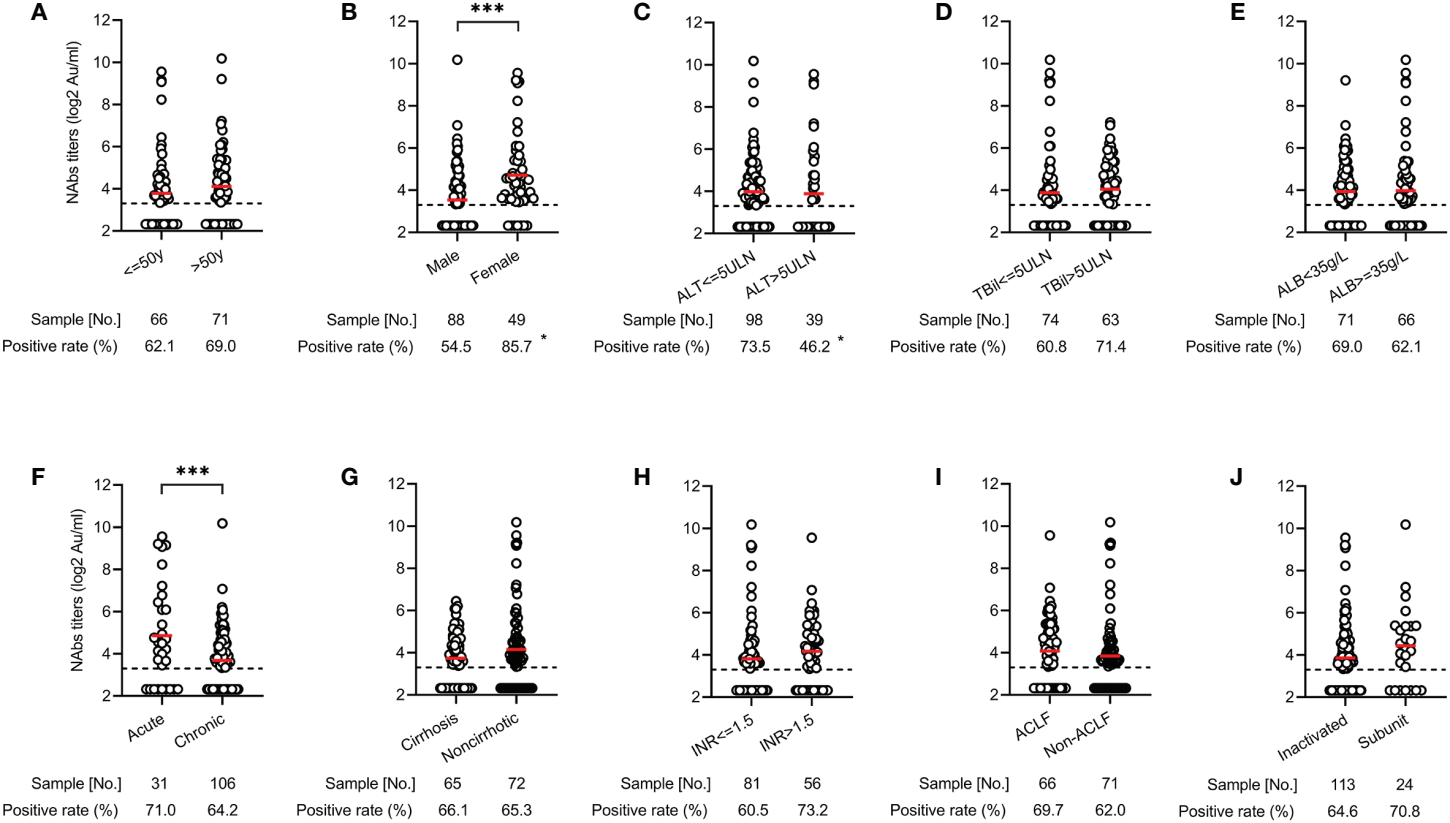
Distribution of antibody responses after COVID-19 vaccination across various clinical parameters in patients with liver dysfunction. Comparison of NAbs seropositivity and titers in the different age (≤50 vs >50 years) **(A)**, gender (male vs female) **(B)**, ALT level (≤ 5×ULN vs >5×ULN) **(C)**, total bilirubin level (≤5×ULN vs >5×ULN) **(D)**, albumin level (<35 vs ≥35 g/L) **(E)**, liver disease status (acute vs chronic) **(F)**, cirrhosis (yes vs no) **(G)**, INR value (≤1.5 vs >1.5) **(H)**, ACLF (yes vs no) **(I)**, and vaccine types (inactivated vs subunit recombinant) **(J)**. *<0.05; ***<0.001. ACLF, acute on chronic liver failure; ALT, alanine aminotransferase; COVID-19, coronavirus disease 2019; INR, international normalized ratio; NAbs, neutralizing antibodies; ULN, upper limits of normal.

To investigate risk factors affecting NAbs response, we compared the distribution of seropositivity and titers according to demographics, clinical parameters, and vaccine types. There were no significantly different in seropositivity and titers of NAbs between age (≤50 *vs >*50 years), total bilirubin level (≤5 times the upper limits of normal [ULN] *vs >*5×ULN), albumin level (<35 *vs* ≥35 g/L), cirrhosis (*vs* noncirrhotic), international normalized ratio (INR) value (≤1.5 *vs >*1.5), ACLF (*vs* non-ACLF), and vaccine types (inactivated *vs* subunit recombinant) (all p>0.05) (**Figure 2**). However, both seropositivity (54.5% *vs* 85.7%, p<0.05) and titers (3.46 *vs* 4.76 log_2_AU/ml, p<0.001) of NAbs in males were significantly lower than those in females (**Figure 2B**). Compared with the ALT ≤ 5×ULN group, the seropositivity of NAbs was significantly lower in the ALT > 5×ULN group (46.2% *vs* 73.5%, p<0.05), but there was no difference in the mean titers (3.63 *vs* 4.06 log_2_AU/ml, p>0.05) between two groups (**Figure 2C**). In addition, patients with the chronic liver disease also had lower NAbs titers compared to those with acute liver disease (3.59 *vs* 4.78 log_2_AU/ml, p<0.001) (**Figure 2F**).

Next, we performed multiple regression analysis to assess the association of sex, ALT levels, and liver disease status with NAbs response. After adjusting for potential confounding factors including age, total bilirubin, cirrhosis, ACLF, vaccine types, and interval time, male sex (OR: 0.17; 95% CI: 0.06, 0.46; p<0.001) and more severe liver damage (ALT > 5×ULN) (OR: 0.30; 95% CI: 0.12, 0.71; p<0.01) were significantly associated with reduction of the probability of NAbs seropositivity (**Table 2**). Regarding NAbs titers, male sex (β =-1.18; 95% CI: -1.73, -0.64; p<0.001) and chronic liver diseases (β =-1.45; 95% CI: -2.13, -0.76; p<0.001) were significantly associated with lower NAbs titers (**Table 2**). Our findings showed that male sex, severe liver injury, and chronic liver diseases have increased risk of poor antibody responses in patients with liver damage.

**Table 2:** Multiple regression analysis of risk factors associated with NAbs response to COVID-19 vaccines in patients with liver dysfunction.

Variables	Crude model		Adjusted model	
	OR/β (95% CI)	P value	OR/β (95% CI)	P value
NAbs seropositivity (binary variable)^*^				
Sex (male *vs* female)	0.20 (0.08, 0.49)	<0.001	0.17 (0.06, 0.46)	<0.001
ALT level (>5×ULN *vs* ≤ 5×ULN)	0.31 (0.14, 0.67)	<0.01	0.30 (0.12, 0.71)	<0.01
Chronic liver diseases (yes *vs* no)	0.73 (0.31, 1.75)	0.48	0.39 (0.13, 1.15)	0.09
NAbs titer (continuous variable)^#^				
Sex (male *vs* female)	-1.17 (-1.73, -0.61)	<0.001	-1.18 (-1.73, -0.64)	<0.001
ALT level (>5×ULN *vs* ≤ 5×ULN)	-0.09 (-0.72, 0.54)	0.78	0.02 (-0.61, 0.65)	0.96
Chronic liver diseases (yes *vs* no)	-1.17 (-1.83, -0.52)	<0.001	-1.45 (-2.13, -0.76)	<0.001

^*^Data are presented as OR (95% CI) and p-value. ^#^Data are presented as β (95% CI) and p-value. The crude model adjusts for none. Adjusted model adjust for age (continuous variable), total bilirubin (continuous variable), cirrhosis (binary variable), ACLF (binary variable), vaccine types (binary variable), and interval time (continuous variable). ACLF, acute on chronic liver failure; ALT, alanine aminotransferase; CI, confidence interval; NAbs, neutralizing antibodies; OR, odds ratio; ULN, upper limits of normal.

### 3.4 Impact of artificial liver therapy on seropositivity and antibody titer

**Figure 3:**
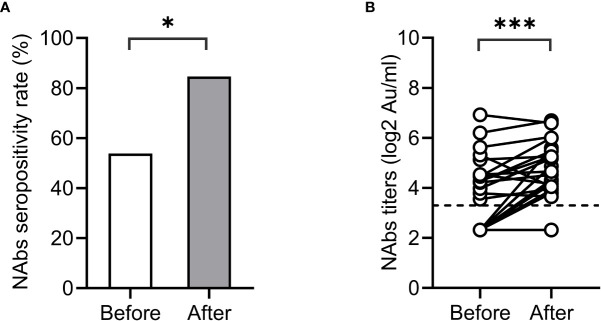
Impact of artificial liver therapy on NAbs levels. NAbs seropositivity **(A)** and titers **(B)** before and after artificial liver therapy in patients with liver failure.*<0.001.

The artificial liver blood purification system is an important treatment for patients with liver failure. It is unclear whether artificial liver therapy will reduce the seropositivity and titer of SARS-CoV-2 antibodies. We conducted pairwise comparisons of the NAbs seropositivity and antibody titers in 26 ACLF patients before and after artificial liver plasmapheresis. The characteristics of these patients are listed in **Supplementary Table 1**. Interestingly, the seropositivity and titers of NAbs in ACLF patients did not decrease but increased significantly after artificial liver therapy (before *vs* after: 53.8% *vs* 84.6%, p<0.05; 3.55 *vs* 4.32 log_2_AU/ml, p<0.001) (**Figure 3**).

### 3.5 Sensitivity analysis

To eliminate the potential bias that autoimmune liver disease might introduce to investigating risk factors for NAbs response, we performed a sensitivity analysis. After excluding patients with autoimmune hepatitis, male sex (OR=0.16; 95% CI 0.06, 0.44; p<0.001) and severe liver damage (OR=0.30; 95% CI 0.12, 0.73; p<0.01) were still significantly associated with lower NAbs seropositivity. Male sex (β=-1.21; 95% CI -1.75, -0.66; p<0.001) and chronic liver disease (β=-1.40; 95% CI -2.08, -0.72; p<0.001) also remained associated with lower NAbs titers (**Supplementary Table 2**).

## 4 Discussion

In this study, we investigated the effect of liver injury on antibody responses following prior COVID-19 vaccinations and reported three key findings (1): compared with healthy people, the seropositivity and titer of NAbs in patients with liver dysfunction were significantly lower and the titer decreased faster, (2) male sex, severe liver injury and chronic liver diseases have increased risk of poor antibody responses, and (3) artificial liver treatment does not reduce NAbs titers in patients with liver failure.

Most of the patients with liver damage in this study had chronic liver disease, and the study found that the seropositivity and titer of NAbs after COVID-19 vaccination in patients with liver damage were significantly lower than those in healthy subjects. Similar to the results of previous studies (9–12), our research also suggested that the antibody response rate and titer after COVID-19 vaccination in patients with chronic liver disease were reduced, which might be related to the basis of immune impairment in patients with chronic liver disease. This view is also supported by the significantly increased rates of antibody non-response in patients with immunosuppressive liver transplantation, autoimmune liver disease, and patients with systemic diseases mediated by immune disorders (13–16).

In this study, male sex, severe liver injury, and chronic liver diseases were found to be risk factors for poor antibody response. In the phase 3 randomized controlled trial of an inactivated vaccine, antibody response rates were lower in healthy male volunteers than in women (17). Zheng (18) and other studies (19) also found that female participants developed higher titers of NAbs compared to the male sex. In a study of a population with cirrhosis, Zhang and his colleagues (12) found that Child-Pugh score of B and C levels is associated with hyporesponsive to COVID-19 vaccination. However, in this study, the presence of cirrhosis was not found to be associated with either seropositivity or titer of NAbs, possibly due to the relatively small number of patients with cirrhosis observed in our study.

The artificial liver system is an important treatment method for severe liver function injury, especially liver failure, among which plasma exchange and plasma adsorption are the most commonly used modes (20). In some autoimmune diseases, such as systemic lupus erythematosus, plasma exchange, and plasma adsorption can reduce the titer of circulating antibodies in patients to achieve the purpose of disease control (21–23). It is still unclear whether artificial liver therapy will reduce COVID-19 antibody titers in patients with chronic liver disease who have received the COVID-19 vaccine when liver function is severely impaired and liver failure occurs due to disease progression. In this study, in 26 patients with liver failure, the titer of NAbs in patients with artificial liver treatment did not decrease compared with that before treatment. Interestingly, most patients had significantly higher titers of NAbs than before treatment. This phenomenon may reveal that these patients received a large amount of serum from healthy volunteers, while healthy adults in China have extremely high rates of COVID-19 vaccination and antibody response. The duration of changes in antibody titers associated with artificial livers requires further follow-up studies.

Several previous studies have suggested that patients with diseases mediated by immune disorders have significantly reduced antibody responses to COVID-19 vaccines (15, 16). In a prospective observational study including patients with autoimmune liver disease, the use of immunosuppressive agents increases the risk of poor antibody response to inactivated COVID-19 vaccine in AILD patients (14). Considering the potentially negative effects of immunosuppressive agents using in patients with autoimmune hepatitis, we performed a sensitivity analysis after excluding these patients. The results showed that male sex, severe liver damage, and chronic liver disease were still associated with poor antibody responses to the COVID-19 vaccines. These results indicated that our conclusions are robust and reliable.

There are some shortcomings in this study. First, the number and duration of longitudinal follow-up of participants were relatively insufficient. Second, the effects of medications on patients were not analyzed, such as statins. Previous studies have shown that statins can reduce influenza vaccine response (24). Finally, although we adjusted for some important confounding variables (eg, age, vaccine types, interval times, etc.) to make the results reliable, it is undeniable that there are still some confounding factors that were not included.

In conclusion, this study provides evidence for poor response to the COVID-19 vaccines in patients with liver dysfunction. Especially in males, those with chronic liver disease and severe liver injury, the NAbs response will be significantly reduced, resulting in less protective effect.

## Author contributions

HL, SL, YL and SZ contributed to the conception and design, analysis, interpretation of the data, and critical revision of important intellectual content. PX, XW and HD collected the data and did the analysis. All authors approved the final version and agreed to be accountable for all aspects of the work. All authors contributed to the article and approved the submitted version.
