# Supplementary material for: Analysis of neutralizing antibodies to COVID-19 inactivated or subunit recombinant vaccines in hospitalized patients with liver dysfunction
Source: Front Immunol. 2023 Jan 18;14:1084646. doi: 10.3389/fimmu.2023.1084646 (PMC9889857; doi:10.3389/fimmu.2023.1084646)
Supplement: Supplementary file 1 [file DataSheet_1.docx]

**Supplementary Table 1.** Baseline characteristics of patients with ACLF receiving artificial liver therapy.

| **Variables** | **Patients (n=26)** |
| --- | --- |
| Age (years), mean (SD) | 48.0 (13.0) |
| Male, n (%) | 17 (65.4) |
| White blood cell (10^3^/μL), median (IQR) | 5.85 (4.21-7.79) |
| Hemoglobin (g/L), median (IQR) | 115.0 (94.0-127.0) |
| Platelet (10^3^/μL), median (IQR) | 90.0 (56.0-118.0) |
| ALT (U/L), median (IQR) | 126.0 (57.5-348.0) |
| AST (U/L), median (IQR) | 120.0 (65.5-292.5) |
| ALP (U/L), median (IQR) | 131.0 (95.5-170.5) |
| GGT (U/L), median (IQR) | 86.0 (48.5-139.5) |
| Total bilirubin (μmol/L), median (IQR) | 279.8 (222.3-395.1) |
| Albumin (g/L), mean (SD) | 32.6 (3.6) |
| INR, median (IQR) | 2.2 (1.8-2.8) |
| Prothrombin time, median (IQR) | 24.4 (20.4-34.4) |
| Vaccine type, n (%) |  |
| Inactivated | 22 (84.6) |
| RBD-subunit recombinant | 4 (15.4) |

Data are presented as mean (SD), median (IQR), or n (%). ACLF, acute on chronic liver failure; ALT, alanine aminotransferase; ALP, alkaline phosphatase; AST, aspartate aminotransferase; GGT, gamma-glutamyl transferase; INR, international normalized ratio; IQR, interquartile range; RBD, receptor-binding domain; SD, standard deviation.

**Supplementary Table 2.** Sensitivity analysis^*^.

| **Variables** | **Crude model** | |  | **Adjusted model** | |
| --- | --- | --- | --- | --- | --- |
|  | **OR/β (95% CI)** | **P value** |  | **OR/β (95% CI)** | **P value** |
| **NAbs seropositivity (binary variable)** ^#^ | | | | | |
| Sex (male vs female) | 0.19 (0.08, 0.47) | <0.001 |  | 0.16 (0.06, 0.44) | <0.001 |
| ALT level (>5×ULN vs ≤ 5×ULN) | 0.29 (0.13, 0.64) | <0.01 |  | 0.30 (0.12, 0.73) | <0.01 |
| Chronic liver diseases (yes vs no) | 0.73 (0.30, 1.77) | 0.49 |  | 0.37 (0.12, 1.11) | 0.08 |
| **NAbs titer (continuous variable)** ^&^ | | | | | |
| Sex (male vs female) | -1.18 (-1.77, -0.60) | <0.001 |  | -1.21 (-1.75, -0.66) | <0.001 |
| ALT level (>5×ULN vs ≤ 5×ULN) | -0.14 (-0.79, 0.52) | 0.69 |  | 0.03 (-0.61, 0.67) | 0.93 |
| Chronic liver diseases (yes vs no) | -1.17 (-1.84, -0.49) | <0.001 |  | -1.40 (-2.08, -0.72) | <0.001 |

^*^ Patients with autoimmune hepatitis were excluded from the analysis.

^#^ Data are presented as OR (95% CI) and p-value. ^&^ Data are presented as β (95% CI) and p-value. The crude model adjusts for none. Adjusted model adjust for age (continuous variable), total bilirubin (continuous variable), cirrhosis (binary variable), ACLF (binary variable), vaccine types (binary variable), and interval time (continuous variable). ACLF, acute on chronic liver failure; ALT, alanine aminotransferase; CI, confidence interval; NAbs, neutralizing antibodies; OR, odds ratio; ULN, upper limits of normal.
